# Creating a medical dictionary using word alignment: The influence of sources and resources

**DOI:** 10.1186/1472-6947-7-37

**Published:** 2007-11-23

**Authors:** Mikael Nyström, Magnus Merkel, Håkan Petersson, Hans Åhlfeldt

**Affiliations:** 1 Department of Biomedical Engineering, Linköpings universitet, SE-58185 Linköping, Sweden; 2 Department of Computer and Information Science, Linköpings universitet, SE-58183 Linköping, Sweden

## Abstract

**Background:**

Automatic word alignment of parallel texts with the same content in different languages is among other things used to generate dictionaries for new translations. The quality of the generated word alignment depends on the quality of the input resources. In this paper we report on automatic word alignment of the English and Swedish versions of the medical terminology systems ICD-10, ICF, NCSP, KSH97-P and parts of MeSH and how the terminology systems and type of resources influence the quality.

**Methods:**

We automatically word aligned the terminology systems using static resources, like dictionaries, statistical resources, like statistically derived dictionaries, and training resources, which were generated from manual word alignment. We varied which part of the terminology systems that we used to generate the resources, which parts that we word aligned and which types of resources we used in the alignment process to explore the influence the different terminology systems and resources have on the recall and precision. After the analysis, we used the best configuration of the automatic word alignment for generation of candidate term pairs. We then manually verified the candidate term pairs and included the correct pairs in an English-Swedish dictionary.

**Results:**

The results indicate that more resources and resource types give better results but the size of the parts used to generate the resources only partly affects the quality. The most generally useful resources were generated from ICD-10 and resources generated from MeSH were not as general as other resources. Systematic inter-language differences in the structure of the terminology system rubrics make the rubrics harder to align. Manually created training resources give nearly as good results as a union of static resources, statistical resources and training resources and noticeably better results than a union of static resources and statistical resources. The verified English-Swedish dictionary contains 24,000 term pairs in base forms.

**Conclusion:**

More resources give better results in the automatic word alignment, but some resources only give small improvements. The most important type of resource is training and the most general resources were generated from ICD-10.

## Background

Medical terminology systems are needed to support manual and automatic data processing in the health care information systems. If the same terminology systems are going to be used in countries with different languages the systems need to be translated, but manual translations are expensive.

Collections of texts in at least two languages in parallel have been used for creating dictionary-like data for reuse in new translations since the beginning of the nineties[1]. A possibility of reducing the costs for translation of medical terminology systems is therefore to use word alignment on already translated systems to generate resources for translation of other systems. The rubrics in the medical terminology systems have a highly repetitive structure in comparison with natural language texts, which are normally used in word alignment methods. The quality of the alignment can therefore be expected to be higher than normal when medical terminology systems are used as sources.

In an earlier study we used interactive word alignment to generate a medical English-Swedish dictionary from a collection of the medical terminology systems ICD-10, ICF, MeSH, NCSP and KSH97-P[2]. With the best configuration we were able to recognise term pairs for the dictionary from the collection with recall of 0.77 and precision of 0.76, but we hypothesized we could exploit the highly repetitive structure in the rubrics to achieve even better results. An interesting analysis is therefore to explore how linguistic standard methods best can be used on medical terminology systems with their particular content and structure.

### Objective

In automatic word alignment parallel text sources were aligned using different types of resources. The parallel text sources we used in this study were terminology systems available in English and Swedish. The types of resources we used were static resources, such as standard dictionaries and parts-of-speech correspondences, statistical resources, such as statistically derived dictionaries, and training resources, such as information acquired during manual word alignment of the parallel text sources. In this study we evaluated how different resources influenced performance according to recall and precision, and whether a specific resource was important or not. Moreover, we studied similarities and differences inside and among the terminology systems. We accomplished this by altering the resources and medical terminology systems we used. The measured similarities and differences were mainly based on whether the used words (contents) in the rubrics were the same, whether the structures of the rubrics were repetitive and whether the structures in the translations were changed according to the original.

Our word alignment resulted in a list of candidate term pairs for an English-Swedish dictionary tailored for the medical terminology systems domain. Finally, we evaluated the quality of the generated candidate term pairs.

### Word alignment

Word alignment can be described as the task of finding correspondences on the word or phrase level between a source text and its translation. If languages were structured in identical ways across language borders, this task would be relatively simple as it would mainly entail finding one-to-one correspondences, that is, instances where one word translates exactly to one word. The task is however more complicated as term equivalents come in both single-word units and multi-word units and there are no straightforward mapping techniques.

The standard approaches to word alignment are of two main strands[3]

1. statistical (or estimation) approaches

2. linguistic approaches

The statistical approaches use probabilistic translation models estimated from parallel corpora. Practically all work within statistical word alignment stems from the early works in statistical machine translation done by Brown et al.[4].

The linguistic approach originates from early work in lexicography where parallel corpora were used to create bilingual lexicons and to find support for how to disambiguate lexical items with the help of parallel linguistic data. Linguistic approaches often use rules for segmentation into lexical units, bilingual dictionaries and rules on word order and positions, as well as rules on corresponding parts-of-speech labels.

As linguistic approaches often use some kind of statistical association measures such as t-scores or the Dice coefficient, they are often regarded not only as linguistic but also as hypothesis testing approaches[5]. This is because there is more than one resource contributing to finding the correct alignment. The challenge in automatic word alignment lies in finding the right resources and to combine them in the right way to obtain optimal alignment results.

Word alignment systems can be used in several application areas, for example, to create bilingual dictionaries used in lexicographical work, bilingual terminology for translators, machine-readable lexicons to be plugged into machine translation systems and in translation studies, to be able to study relationships between originals and translations. The primary usefulness of word alignment is that it will recover and present candidate term pairs from a potentially previously unknown domain by means of an analysis of a text corpus.

## Methods

### Overview

We extracted the rubrics existing in parallel in both English and Swedish in the terminology systems ICD-10, ICF, MeSH, NCSP and KSH97-P and used the rubrics as input data to the automatic word alignment. We then performed an evaluation and divided the rubrics into the partitions described in Table 1.

We performed automatic word alignment where we used static resources, statistical resources and training resources are used. One type of static resource is the 'standard resources', which is two standard English-Swedish dictionaries and pattern resources for common parts-of-speech correspondences. The other two types of static resources are a parts-of-speech blocker and the MeSH-dictionary created from one-word rubrics in MeSH. The statistical resources are dictionaries generated automatically by statistical analysis of the parallel texts of the different partitions. The training resources are built from manual word alignment form a sample of the different partitions.

In the automatic word alignment we altered the used resources according to the batches presented in Table 2, 3, 4, 5, 6, 7. Each batch had a specific configuration according to a specific issue. We calculated recall, precision and F-score for the automatic word alignment in comparison with a manually word aligned sample from the different partitions.

The automatic word alignment also produced English-Swedish candidate term pairs and we manually evaluated the candidate term pairs and collected the correct term pairs to an English-Swedish medical dictionary.

We also measured the intra-rater reliability using the F-score.

A glossary is included in Appendix1.

### Alignment tools

For this experiment we use word alignment software from the ITools suite developed at Linköping University and at Fodina Language Technology. The ITools suite consists of a range of tools with the ultimate goal of producing standardised term banks from parallel texts or translation memories [6-8]. The ITools suite consists of pre-alignment components that handle morphosyntactic tagging, statistical processing and a training phase involving manual and interactive word alignment. After the automatic alignment has taken place, the results are further processed in the post-alignment stage. For a thorough description of the terminology extraction process and standardisation process, see [5].

**Figure 1:**
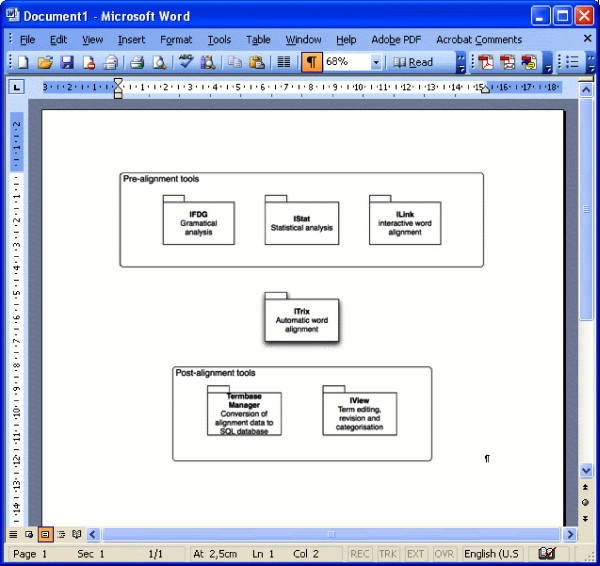
**The ITools suite**. The heart of the ITools suite is the ITrix application which performs the automatic word alignment. The other tools used are three tools applied before ITrix in the pre-alignment phase: IFDG, for tagging, IStat, for statistical processing, and ILink, for training and creating resources used by ITrix. After the word alignment, the candidate term pairs are converted into an SQL database by Termbase Manager, and the candidate term pairs can be revised graphically in the IView application.

The most important parts of the ITools suite are the following six software components (see also Figure 1).

• **IFDG **– a front-end to Connexor's Machinese Syntax syntactic parsers, formerly known as Functional Dependency Grammar parsers[9]. Currently, Machinese Syntax supports ten European languages and provide data of the following kinds for each word token: 1) Base form (lemma), 2) Parts-of-speech and morphological features 3) Syntactic function, 4) Dependency relation and head of dependency relation. The Machines Syntax parser is designed for standard English and Swedish and is not tailored towards specific domains such as medicine. This means that for both Swedish and English there are problems with medical rubrics with Latin and Greek origins as the rule-based morphological component have problems to identify the correct lemma of inflected words.

• **IStat **– a statistical tool that creates bilingual lexical resources from the source and target text, based on co-occurrence measures. Bilingual resources derived by various association measures and by using the GIZA++ toolkit [2] can be created using IStat.

• **ILink **– an interactive word aligner. This tool is used for training the automatic word aligner. Bilingual resources are being built up incrementally each time words and phrases are aligned in a graphical user interface. A user/annotator is in control of the alignment process and confirms, rejects or modifies the alignments proposed by ILink. The created resources are then fed into the automatic word aligner.

• **ITrix **– a fully automatic word aligner. The source and target texts are word aligned automatically using the resources created by ILink and static resources such as bilingual lexicons and pattern resources. The automatic alignment consists of a voting procedure, where each resource, be they static or dynamic, votes for different alignment combinations, in each sentence pair. The sentences in a sentence pair are represented as a matrix where the source sentence builds the horizontal axis and the target sentence the vertical axis. In the first step, only true points of correspondence (TPCs), highly probable single word correspondences, are established a similar way to Melamed[10]. These TPCs are then expanded, if possible, in the matrix into multi-word units by joining adjacent cells according to the voting values. ITrix can be said to be a hypothesis testing application where linguistic types of data (e.g. parts-of-speech patterns) are utilised together with statistically derived data.

• **Termbase Manager **– a conversion utility that transforms the results from the automatic alignment into a database, containing candidate term pairs, inflectional variants, grammatical information, examples, etc.

• **IView **– a graphical interface to the SQL database. In IView the terminologist can filter, search, revise and categorise all candidate term pairs and finally export the generated dictionary to an external file format, such as TBX, MultiTerm, OLIF or Excel spreadsheets.

A more detailed description of the workflow when going from parallel texts to bilingual standardised terminology banks using the ITools suite is described in [6].

The focus in this project lies on the use of different resources and strategies in automatic alignment. It is important to distinguish between three types of resources

1. Static resources. These are resources that are fixed, such as general bilingual dictionaries or domain-specific bilingual dictionaries, for example an English-Swedish medical dictionary. Static resources can also contain fixed rules for parts-of-speech correspondences.

2. Statistical resources. These resources contain statistical data from the source and target texts, for example, statistically derived bilingual lexicons from the specific source and target texts.

3. Dynamic resources. These resources are built up during training of a specific training set belonging to the same domain as the alignment project texts.

One could say that the *static resources *are independent from the alignment project in the sense that there is nothing in a static resource that is directly derived from the alignment project texts. The statistical resources are typically run once at an initial stage of the project, and the resources never change after that. The dynamic resources, however, continue to grow depending on the extent of the training efforts.

The *dynamic resources *are intended to reflect the specific characteristics of the material that is being aligned. The dynamic resources are built by manual and interactive training on four levels simultaneously, namely for word form, lemma form, parts-of-speech and syntactic function levels. This means that training on sentence pairs will result in dynamically created lexicons containing both word forms (inflections) and lemmas (base forms) as well as data on parts-of-speech and function correspondences from those sentences. These dynamic resources are what we refer to as *training resources *for a given set of documents. Training should be done in a consistent manner, i.e., the same principles for building the training resources should be applied over all the training material, because inconsistent training will result in decreased performance of the automatic alignment.

The *standard resources *used in this project consist of two static standard dictionaries containing a total of 22,500 English-Swedish entries. These dictionaries are considered to be non-medical and non-technical and are used as general resources to guide the alignment and to determine what is domain specific terminology and what is not. Also included in the standard set-up are pattern resources for common parts-of-speech correspondences, for example that two nouns in English often correspond to one noun in Swedish (NN#N), as well as standard punctuation patterns that are applicable in general alignment between English and Swedish.

In the pre-alignment phase, it is possible to run a statistical analysis on the parallel text to create *statistical resources*. The ITools suite can use such statistically derived dictionaries in different fashions such as co-occurrence measures like t-score and the Dice coefficient. In this project, the GIZA++ tool kit was used to generate probabilistic dictionaries[3] of two kinds, for the lemma level and on the word form level.

A *parts-of-speech blocker *is also used in this experiment. This blocker uses the parts-of-speech tag provided by IDFG and is devised so that it will filter out any alignment that matches a certain predefined pattern that is deemed to be unwanted in this domain. For example, the regular expression

.+#(Cc|Interp|Det|Cs|Pron) .+

will block out any alignment where the target side (Swedish) contains a multi-word unit that starts with a conjunction, punctuation, article, subjunction or pronoun. Instead the next best alignment will be chosen. For example, if the highest ranking nominee term pair is 'high blood pressure – ett högt blodtryck', where the Swedish indefinite article 'ett' starts the Swedish nominee term, then this nominee term will be blocked by the pattern above.

Even when none of the resources mentioned above are used in the alignment set-up a few simple heuristics are still applied, such as a cognate test (where similar strings are rewarded) and a strategy that the same parts-of-speech category in both the source and target candidate contribute to alignment preferences.

### Ranking and filtering candidate term pairs

In any word alignment project it is desirable to optimize the quality of the aligned data by stripping away poor quality alignments and keeping the high quality ones as this will leave less manual work in the actual standardization process. To achieve this, one has to order the candidate term pairs in, for example, descending quality order. Ordering candidate term pairs can be done using different metrics. One such metric that has been used in term extraction research is the Dice coefficient of association[11]. A common approach in applying Dice coefficient as a ranking metric is to collect corpus statistics[12]. The metric used here is the qvalue, a metric specifically designed to operate on aligned data[13].

The input data used are all available in the SQL database, which contains information such as

• Type Pair Frequencies, TPF, i.e. the number of times where the source and target types are aligned

• Target types per Source type, TpS, i.e. the number of target types a specific source type has been aligned to. For example, if the source type A is aligned to the target types B and C, two type pairs exist, A-B and A-C. For both these type pairs, the TpS value is 2.

• Source types per Target type, SpT, i.e. the number of source types a specific target type has been aligned to. Given the example provided to explain the TpS, the SpT values for the two type pairs would be 1 for A-B, and 1 for A-C. This means that low SpT and TpS values correspond to consistent usage of target and source types if the aligned data are fairly correct.

Using this information, we can describe the qvalue in the following way

$$Q-value=\frac{Type\;Pair\;Frequencies}{Target\;types\;per\;Source\;type+Source\;types\;per\;Target\;type}$$

In short, one can describe the qvalue as a metric that assigns high qvalues for candidate term pairs with consistent translations and high frequencies, whereas low qvalues indicate candidate term pairs with inconsistent usages and low frequencies. The qvalue metric could be applied either to base forms (lemmas) or to word forms (inflections). In previous alignment projects, it has been shown that a qvalue threshold at 0.4 will yield precision rates for candidate term pairs of more than 90 per cent [6,13]. To illustrate the application of qvalues, consider a candidate term pair like 'disease – sjukdom' which has a type pair frequency of 630 in the alignment results. Given that 'sjukdom' has 18 source candidates (SpT is 18) and that the English 'disease' occurs in 48 distinct pairs (TpS is 48), the qvalue for this term pair is 9.54 derived from 630/(48+18). This can be compared to a very poor alignment, for example 'disease – sjuk', which with a frequency of 1 will yield a qvalue of 0.0196 derived from 1/(48+3), given that 'disease' occurs in 48 and 'sjuk' in 3 different candidate term pairs.

A unique term pair occurrence will always yield the qvalue 0.5 if the respective source and target term is not aligned in any other pair. This means that it can be expected that medical rubrics will produce a high number of candidate term pairs with the qvalue 0.5, because of the specific characteristics of such input data, i.e. a high number of unique entries.

### Terminologies

The terminologies we use in this study are the five medical terminology systems ICD-10, ICF, MeSH, NCSP and KSH97-P, which are further described in Appendix2. We extracted all the rubrics which exist in parallel in both English and Swedish together with their code. In this case rubric means the label associated with each code. When both a preferred rubric and synonym rubrics exist we included only the preferred rubric. A rubric pair example is the English rubric 'Enteropathogenic Escherichia coli infection' and the Swedish rubric 'Infektion med tarmpatogena Escherichia coli-bakterier' accompanying the ICD-10 code A04.0.

### Terminology partitions

#### Manual inspection

In the earlier study [2] we had an indication that parts of the medical terminology systems worsen instead of improve the quality of the resources generated from the terminology systems. The author MN therefore performed a manual inspection of the terminology systems to find subparts from the terminology systems that have different characteristics than the main part of the systems. Three such subparts were found

• In ICD-10 chapter2 level4 the Swedish rubrics frequently start with phrases like 'Malign tumör' ('Malignant neoplasm') and 'Benign tumör' ('Benign neoplasm'), but these start phrases do not have translational correspondences in the English rubrics. Instead this information is implied from the super-ordinate rubrics. An example is ICD-10 code C38.0 with the English rubric 'Heart' and the Swedish rubric 'Malign tumör i hjärtat' (literally 'Malignant neoplasm of heart').

• In NCSP chapterN the translations of the rubrics are more paraphrased than in the rest of NCSP. An example is NCSP code NCB09 with the English rubric 'Primary partial prosthetic replacement of elbow joint not using cement; other or unspecified' and the Swedish rubric 'Primär halv- eller delprotes i armbågsled utan cement' (literally 'Primary half or part prothesis in elbow joint without cement').

• In MeSH the rubric pairs where at least one rubric consists of one word act as a kind of English-Swedish dictionary. This is because they consist of rubrics that can not be divided into smaller parts. Some examples are MeSH code D000001 with the English rubric 'Calcimycin' and the Swedish rubric 'Kalcimycin' (literally 'Calcimycin'), D000002 with the English rubric 'Temefos' and the Swedish rubric 'Temefos' (literally 'Temefos'), and D000003 with the English rubric 'Abattoirs' and the Swedish rubric 'Slakthus' (literally 'Abattoirs').

#### Statistical evaluation

When the numbers of words or numbers of characters in corresponding English and Swedish rubrics show differences that are not systematic, this indicates that the translation process have made insertions or deletions in the translated rubrics. We suspected these kinds of alterations as sources of errors when automatic word alignment resources are generated.

To confirm if ICD-10 chapter2 level4 and NCSP chapterN had different numbers of words and/or numbers of characters than the remainders of the respective terminology systems, we compared the length of the rubrics by correlation, rubric ratio and rubric ratio resampling. These comparisons are described below.

In the correlation analysis we compared corresponding rubrics to each other both according to number of words and according to number of characters. Since the MeSH subpart with one-word rubrics is produced through filtering, correlation is not a relevant measure. Consequently we only included the other subparts in the correlation analysis. We used Kendall's tau-b correlation method and the results are included in Table 1. The results confirm that there are differences between ICD-10 chapter 2 level 4 and NCSP chapterN and the remainders of the respective terminology system.

**Table 1:** Partition characteristics

Partition	Content	Word correlation	Character correlation	Word ratio difference	Rubrics	English rubric average number (standard deviation) of words	Swedish rubric average number (standard deviation) of words	English unique words	Swedish unique words	English unique words per rubric	Swedish unique words per rubric
All	All terminology systems	0.78	0.79		38,575	3.7 (2.9)	3.3 (3.0)	17,679	25,848	0.5	0.7
1	MeSH, one word in either English or Swedish rubric			0.56	13,514	1.5 (0.7)	1.0 (0.1)	11,267	13,581	0.8	1.0
2	MeSH, more than one word in both English and Swedish rubrics	0.52	0.71	0.30	5,568	2.6 (0.8)	2.3 (0.7)	5,434	6,443	1.0	1.2
3	ICF, whole	0.69	0.79	0.53	1,496	4.7 (2.5)	4.2 (2.8)	991	1,263	0.7	0.8
4	KSH97-P, whole	0.70	0.67	0.49	968	4.0 (2.5)	3.5 (2.4)	1,324	1,382	1.4	1.4
5	ICD-10, except chapter 2 level 4	0.77	0.75	0.37	10,791	5.2 (3.0)	5.2 (3.4)	5,144	7,219	0.5	0.7
6	NCSP, except chapter N	0.64	0.63	0.38	4,137	5.8 (2.7)	5.0 (2.5)	1,758	2,347	0.4	0.6
7	ICD-10, chapter 2 level 4	0.38	0.45	0.71	713	3.6 (2.2)	6.3 (2.7)	443	535	0.6	0.8
8	NCSP, chapter N	0.55	0.48	0.25	1,388	9.4 (2.6)	7.7 (2.3)	249	285	0.2	0.2

Content of the partitions.

Kendall's tau-b correlation between the English rubrics and corresponding Swedish rubrics according to number of words and number of characters and average absolute differences between the ratio for all rubrics in the partition and the grand mean of the different terminology partitions.

Number of parallel rubrics, average number and standard deviation of words per rubrics, number of unique words, and number of average unique words per rubric of the different terminology partitions.

Compared to English, Swedish is a much more compound-rich language. The difference in absolute number of words between the corresponding English and Swedish rubrics is therefore not a completely useful measure. Instead we analysed the ratio between the numbers of words in corresponding rubrics.

In the rubric ratio analysis we calculated the grand mean ($\bar{q}$) of the number of words in the English rubrics (***e***) and the number of words in the corresponding Swedish rubrics (***s***) for all rubrics (***n***). The result was 1.29. This means that for each word in a Swedish rubric 1.29 words can be expected in the corresponding English rubric.

$$\bar{q}=\frac{\sum_{k=1}^{n}\frac{e_{k}}{s_{k}}}{n}$$

For each partition (***p***) the average absolute differences ($\bar{\Delta q_{p}}$) between the ratio for all rubrics and the grand mean ($\bar{q}$) were then calculated.

$$\bar{\Delta q_{p}}=\frac{\sum_{k=1}^{n_{p}}\left|\frac{e_{p_{k}}}{s_{p_{k}}}-\bar{q}\right|}{n_{p}}$$

The results are included in Table 1. As can be seen there is a relatively large difference between ICD-10 chapter 2 level 4 and the rest of ICD-10, which confirms that the problem with insertions of start phrases in chapter2 level4 makes difference. The difference between NCSP chapterN and the rest of NCSP is also noticeable and chapterN has the lowest ratio of the evaluated parts. This finding supports the manual inspection finding that NCSP chapterN is translated in a different manner than the main part of NCSP.

In the rubric ratio resampling analysis we put together the two parts of ICD-10 in one set and calculated the average absolute difference ratio for the whole ICD-10. The result was 0.39. We extracted a random subset containing the same number of rubric pairs as chapter2 level4 10,000 times. In 95 per cent of the cases the average absolute difference ratio were between 0.37 and 0.42. This is a considerably lower result than the result for chapter2 level4 alone, which was 0.71. This finding supports that chapter2 level4 is translated in a different manner than the main part of ICD-10.

In the same way we carried out a resampling analysis with NCSP. Here the average absolute difference ratio for the whole NCSP was 0.35 and the interval between 0.33 and 0.37. This is a considerably higher result than the result for chapterN alone, which was 0.25. This finding supports that NCSP chapterN is translated in a different manner than the main part of NCSP.

#### Partition division and statistics

In line with the findings in the manual inspection and statistical evaluation we divided the English and Swedish terminology system rubrics into 8 partitions, which are described in Table 1. We ordered the partitions according to the assumed quality of the automatic word alignment resources which could be generated from the partitions. We assumed that partition 1 to give the highest quality and partition 8 the lowest. However, the order of the partitions was not critical for the study. This order is described in Table 1. To acquire more information about each partition we calculated more descriptive statistics, which are included in Table 1.

### Performance measures

In this study we used the performance measures recall, precision and F- score. We calculated the measures by comparing automatic word alignment and manual word alignment of test sets where the quality of the automatic alignment is evaluated and the manual alignment is the gold standard. The formulas used for calculating recall, precision and F-score are described below.

$$recall=\frac{automatic\;alignments\cap manual\;alignments}{manual\;alignments}$$

$$precision=\frac{automatic\;alignments\cap manual\;alignments}{automatic\;alignments}$$

$$F\text{-}score=\frac{2*recall*precision}{recall+precision}$$

#### Intra-rater reliability

In the intra-rater reliability study we compared two manual word alignments by the F-score. We selected the F-score because of its dependence on recall and precision and because of the symmetry in the F-score formula, which eliminate the need to select one of the alignments as a gold standard.

### Experiment set-up

We started the experiments with preparation and the author MN manually word aligned the terminology system rubrics in the training set. Another author, MM, carried out the automatic word alignments in 6 batches with different configurations to be able to evaluate the alignments. We generated a list of candidate term pairs for an English-Swedish dictionary and MN manually evaluated the list. MN once again carried out the manual word alignment in order to measure the intra-rater reliability.

#### MeSH-dictionary

In the manual inspection of the terminology systems we found that partition 1 act as a kind of English-Swedish dictionary. Therefore there was no need to word aligning partition 1 and we therefore left out this partition from the word alignment. Instead the partition was used as a static resource called MeSH-dictionary in the word alignment process.

### Manual word alignment

We pre-processed partitions 2–8 for the word alignment. From these partitions we randomly sampled 5 percent to a training set and 5 percent to a test set. The author MN then manually aligned the training sets and test sets following the word alignment style guide included in Appendix3. We used the training sets to build training resources for the automatic word alignment and saved the test sets for later use as a gold standard for the automatic word alignment.

### Automatic word alignment

In the automatic word alignment the author MM automatically aligned the test sets from the partitions 2–8 in 6 batches. Each batch was constructed to answer one or two questions.

In the batches we altered the partitions used for building the resources for the automatic word alignments, the different kinds of resources used in the automatic word alignments and the partitions which were automatically word aligned. We then compared the results from the automatic word alignment with the manual word aligned test sets and calculated figures for recall, precision and F-score.

We generated a list of candidate term pairs by automatic word alignment of the whole content (partition 2–8).

#### Common configurations

The three most commonly used configurations in this study were

• 'CfStatistical', where only statistical resources, the statistically generated dictionaries, were used as resources.

• 'CfStatisticalStatic', where static resources such as standard resources, parts-of-speech blocker and MeSH-dictionary together with the resources from the configuration CfStatistical were used.

• 'CfStatisticalStaticTraining', where the training resources from the manual word alignment together with the resources from the configuration CfStatisticalStatic were used.

These configurations were selected as most interesting because CfStatistical measure the performance of a purely statistical system, CfStatisticalStatic measure the performance of a system without training data and CfStatisticalStaticTraining measure the performance of the complete system. Other configurations had been interesting, but were left out to limit the scope of the study.

##### Batch 1

Batch 1 examines the performance of the automatic word alignment for each of the partitions when resources from a single partition are used to align the same partition. The performance also indicates the similarities and differences inside each partition.

Accordingly, this examination was done with resources used for the automatic word alignment generated from the same partition as the word aligned partition. Each of these runs were aligned with the configurations CfStatistical, CfStatisticalStatic and CfStatisticalStaticTraining. The configurations in this batch are shown in Table 2.

##### Batch 2

Batch 2 examines the performance of the automatic word alignment when generalising resources from one partition to other partitions. The performance also indicates the similarities and differences between a partition and the union of all partitions, which can be seen as an approximation of the medical language in healthcare records.

This examination was done with resources used for the automatic word alignment generated from a single partition and all partitions were automatically aligned. Each of these runs were aligned with the configurations CfStatistical, CfStatisticalStatic and CfStatisticalStaticTraining. The configurations in this batch are shown in Table 3.

##### Batch 3

Batch 3 examines whether the performance of the automatic word alignment improves monotonously when resources from more and more partitions are used or if some partitions worsen the performance when they are included. The performance also indicates if a partition is similar to or different from other partitions.

This examination was done with resources used for the automatic word alignment used cumulatively from the partitions and all partitions were automatically aligned. Each of these runs were aligned with the configurations CfStatistical, CfStatisticalStatic and CfStatisticalStaticTraining. The configurations in this batch are shown in Table 4.

##### Batch 4

Batch 4 examines the performance of the automatic word alignment for each of the partitions when all available resources are used.

This examination was done with resources used for the automatic word alignment generated from all partitions and a single partition was automatically aligned each time. Each of these runs were aligned with the configuration CfStatisticalStaticTraining. The configurations in this batch are shown in Table 5.

##### Batch 5

Batch 5 examines the importance of standard resources, parts-of-speech blocker and MeSH-dictionary for the performance when statistical resources and training resources are used.

This examination was done with resources used for the automatic word alignment generated from all partitions and all partitions were automatically aligned in all runs. Whether the standard resources, the parts-of-speech blocker and the MeSH-dictionary were used or not were altered in all eight possible ways. The configurations in this batch are shown in Table 6.

##### Batch 6

Batch 6 examines the importance of statistical resources and training resources for the performance.

The resources used for the automatic word alignment were generated from all partitions and all partitions were automatically aligned in all runs. The use of static resources, statistical resources and training resources were altered according to Table 7.

#### Candidate term pairs creation

To create candidate term pairs for the dictionary we automatically aligned the whole content of partition 2–8. We used static resources, statistical resources and training resources, because this configuration had shown the best recall and precision in the automatic word alignment of the union of partitions.

### Dictionary creation

We collected all unique candidate term pairs generated for the dictionary creation and grouped them together in their base forms. The author MN then categorised the candidate term pairs as correct term pairs, partly correct term pairs or incorrect term pairs. We considered candidate term pairs already categorised as correct in the earlier study [2] as correct also in the current study and categorised the rest of the candidate term pairs manually. The correct term pairs where then collected to constitute the dictionary.

### Intra-rater reliability

To measure the intra-rater reliability, the same author who manually word aligned the training sets and test sets, MN, realigned the test sets one month after the first alignment. We then compared the test sets aligned on the two occasions and calculated figures for F-score. The author MN also performed a manual inspection to see how the alignments differ. In the inspection each of the corresponding alignments from the two events were compared and the identified differences were analysed.

## Results

### Automatic word alignment

The results of the experiments in batch 1–6 are the recall, precision and F-score figures. These results are presented in Table 2, 3, 4, 5, 6, 7.

**Table 2:** Automatic word alignment results from batch 1

Batch	Run	Resources																	Alignment							Result		
												
		Static			Statistic							Dynamic																
		
		Stan	POS	MeSH	Statistic partition							Training partition							Test partition							Recall	Precision	F-score
								
					2	3	4	5	6	7	8	2	3	4	5	6	7	8	2	3	4	5	6	7	8			
1	1				X														X							0.54	0.56	0.55
1	2					X														X						0.48	0.56	0.52
1	3						X														X					0.38	0.41	0.39
1	4							X														X				0.43	0.48	0.45
1	5								X														X			0.41	0.46	0.43
1	6									X														X		0.40	0.36	0.38
1	7										X														X	0.46	0.52	0.49
1	8	X	X	X	X														X							0.68	0.68	0.68
1	9	X	X	X		X														X						0.72	0.75	0.73
1	10	X	X	X			X														X					0.60	0.60	0.60
1	11	X	X	X				X														X				0.69	0.67	0.68
1	12	X	X	X					X														X			0.65	0.64	0.64
1	13	X	X	X						X														X		0.53	0.48	0.50
1	14	X	X	X							X														X	0.71	0.71	0.71
1	15	X	X	X	X							X							X							0.72	0.70	0.71
1	16	X	X	X		X							X							X						0.80	0.80	0.80
1	17	X	X	X			X							X							X					0.71	0.68	0.69
1	18	X	X	X				X							X							X				0.80	0.78	0.79
1	19	X	X	X					X							X							X			0.83	0.78	0.80
1	20	X	X	X						X							X							X		0.63	0.58	0.60
1	21	X	X	X							X							X							X	0.84	0.85	0.84

Recall, precision and F-score from the automatic word alignment when resources for the automatic word alignment were generated from the same partition as the aligned partition. The configurations CfStatistical, CfStatisticalStatic and CfStatisticalStaticTraining were used.

**Table 3:** Automatic word alignment results from batch 2

Batch	Run	Resources																	Alignment							Result		
												
		Static			Statistic							Dynamic																
		
		Stan	POS	MeSH	Statistic partition							Training partition							Test partition							Recall	Precision	F-score
								
					2	3	4	5	6	7	8	2	3	4	5	6	7	8	2	3	4	5	6	7	8			
2	1				X														X	X	X	X	X	X	X	0.34	0.40	0.37
2	2					X													X	X	X	X	X	X	X	0.34	0.40	0.37
2	3						X												X	X	X	X	X	X	X	0.34	0.40	0.37
2	4							X											X	X	X	X	X	X	X	0.40	0.46	0.43
2	5								X										X	X	X	X	X	X	X	0.35	0.41	0.38
2	6									X									X	X	X	X	X	X	X	0.33	0.40	0.36
2	7										X								X	X	X	X	X	X	X	0.35	0.41	0.38
2	8	X	X	X	X														X	X	X	X	X	X	X	0.60	0.62	0.61
2	9	X	X	X		X													X	X	X	X	X	X	X	0.60	0.62	0.61
2	10	X	X	X			X												X	X	X	X	X	X	X	0.61	0.62	0.61
2	11	X	X	X				X											X	X	X	X	X	X	X	0.65	0.65	0.65
2	12	X	X	X					X										X	X	X	X	X	X	X	0.61	0.63	0.62
2	13	X	X	X						X									X	X	X	X	X	X	X	0.60	0.62	0.61
2	14	X	X	X							X								X	X	X	X	X	X	X	0.61	0.63	0.62
2	15	X	X	X	X							X							X	X	X	X	X	X	X	0.65	0.65	0.65
2	16	X	X	X		X							X						X	X	X	X	X	X	X	0.62	0.63	0.62
2	17	X	X	X			X							X					X	X	X	X	X	X	X	0.64	0.64	0.64
2	18	X	X	X				X							X				X	X	X	X	X	X	X	0.75	0.73	0.74
2	19	X	X	X					X							X			X	X	X	X	X	X	X	0.67	0.67	0.67
2	20	X	X	X						X							X		X	X	X	X	X	X	X	0.64	0.65	0.64
2	21	X	X	X							X							X	X	X	X	X	X	X	X	0.64	0.65	0.64

Recall, precision and F-score from the automatic word alignment when resources for the automatic word alignment were generated from a single partition and all partitions were automatically aligned. The configurations CfStatistical, CfStatisticalStatic and CfStatisticalStaticTraining were used.

**Table 4:** Automatic word alignment results from batch 3

Batch	Run	Resources																	Alignment							Result		
												
		Static			Statistic							Dynamic																
		
		Stan	POS	MeSH	Statistic partition							Training partition							Test partition							Recall	Precision	F-score
								
					2	3	4	5	6	7	8	2	3	4	5	6	7	8	2	3	4	5	6	7	8			
3	1				X														X	X	X	X	X	X	X	0.34	0.40	0.37
3	2				X	X													X	X	X	X	X	X	X	0.41	0.46	0.43
3	3				X	X	X												X	X	X	X	X	X	X	0.46	0.50	0.48
3	4				X	X	X	X											X	X	X	X	X	X	X	0.53	0.55	0.54
3	5				X	X	X	X	X										X	X	X	X	X	X	X	0.57	0.58	0.57
3	6				X	X	X	X	X	X									X	X	X	X	X	X	X	0.59	0.59	0.59
3	7				X	X	X	X	X	X	X								X	X	X	X	X	X	X	0.61	0.61	0.61
3	8	X	X	X	X														X	X	X	X	X	X	X	0.60	0.62	0.61
3	9	X	X	X	X	X													X	X	X	X	X	X	X	0.62	0.63	0.62
3	10	X	X	X	X	X	X												X	X	X	X	X	X	X	0.65	0.65	0.65
3	11	X	X	X	X	X	X	X											X	X	X	X	X	X	X	0.69	0.67	0.68
3	12	X	X	X	X	X	X	X	X										X	X	X	X	X	X	X	0.71	0.68	0.69
3	13	X	X	X	X	X	X	X	X	X									X	X	X	X	X	X	X	0.72	0.69	0.70
3	14	X	X	X	X	X	X	X	X	X	X								X	X	X	X	X	X	X	0.72	0.69	0.70
3	15	X	X	X	X							X							X	X	X	X	X	X	X	0.65	0.65	0.65
3	16	X	X	X	X	X						X	X						X	X	X	X	X	X	X	0.67	0.66	0.66
3	17	X	X	X	X	X	X					X	X	X					X	X	X	X	X	X	X	0.69	0.67	0.68
3	18	X	X	X	X	X	X	X				X	X	X	X				X	X	X	X	X	X	X	0.77	0.74	0.75
3	19	X	X	X	X	X	X	X	X			X	X	X	X	X			X	X	X	X	X	X	X	0.80	0.76	0.78
3	20	X	X	X	X	X	X	X	X	X		X	X	X	X	X	X		X	X	X	X	X	X	X	0.80	0.77	0.78
3	21	X	X	X	X	X	X	X	X	X	X	X	X	X	X	X	X	X	X	X	X	X	X	X	X	0.81	0.77	0.79

Recall, precision and F-score from the automatic word alignment when resources for the automatic word alignment were used cumulatively and all partitions were automatically aligned. The configurations CfStatistical, CfStatisticalStatic and CfStatisticalStaticTraining were used.

**Table 5:** Automatic word alignment results from batch 4

Batch	Run	Resources																	Alignment							Result		
												
		Static			Statistic							Dynamic																
		
		Stan	POS	MeSH	Statistic partition							Training partition							Test partition							Recall	Precision	F-score
								
					2	3	4	5	6	7	8	2	3	4	5	6	7	8	2	3	4	5	6	7	8			
4	1	X	X	X	X	X	X	X	X	X	X	X	X	X	X	X	X	X	X							0.73	0.71	0.72
4	2	X	X	X	X	X	X	X	X	X	X	X	X	X	X	X	X	X		X						0.81	0.81	0.81
4	3	X	X	X	X	X	X	X	X	X	X	X	X	X	X	X	X	X			X					0.81	0.76	0.78
4	4	X	X	X	X	X	X	X	X	X	X	X	X	X	X	X	X	X				X				0.81	0.78	0.79
4	5	X	X	X	X	X	X	X	X	X	X	X	X	X	X	X	X	X					X			0.84	0.79	0.81
4	6	X	X	X	X	X	X	X	X	X	X	X	X	X	X	X	X	X						X		0.74	0.65	0.69
4	7	X	X	X	X	X	X	X	X	X	X	X	X	X	X	X	X	X							X	0.85	0.85	0.85

Recall, precision and F-score from the automatic word alignment when resources for the automatic word alignment were generated from all partitions and a single partition was automatically aligned. The configuration CfStatisticalStaticTraining was used.

**Table 6:** Automatic word alignment results from batch 5

Batch	Run	Resources																	Alignment							Result		
												
		Static			Statistic							Dynamic																
		
		Stan	POS	MeSH	Statistic partition							Training partition							Test partition							Recall	Precision	F-score
								
					2	3	4	5	6	7	8	2	3	4	5	6	7	8	2	3	4	5	6	7	8			
5	1				X	X	X	X	X	X	X	X	X	X	X	X	X	X	X	X	X	X	X	X	X	0.80	0.77	0.78
5	2			X	X	X	X	X	X	X	X	X	X	X	X	X	X	X	X	X	X	X	X	X	X	0.80	0.76	0.78
5	3		X		X	X	X	X	X	X	X	X	X	X	X	X	X	X	X	X	X	X	X	X	X	0.81	0.77	0.79
5	4		X	X	X	X	X	X	X	X	X	X	X	X	X	X	X	X	X	X	X	X	X	X	X	0.81	0.77	0.79
5	5	X			X	X	X	X	X	X	X	X	X	X	X	X	X	X	X	X	X	X	X	X	X	0.81	0.76	0.78
5	6	X		X	X	X	X	X	X	X	X	X	X	X	X	X	X	X	X	X	X	X	X	X	X	0.81	0.77	0.79
5	7	X	X		X	X	X	X	X	X	X	X	X	X	X	X	X	X	X	X	X	X	X	X	X	0.81	0.77	0.79
5	8	X	X	X	X	X	X	X	X	X	X	X	X	X	X	X	X	X	X	X	X	X	X	X	X	0.81	0.77	0.79

Recall, precision and F-score from the automatic word alignment when resources for the automatic word alignment were generated from all partitions and all partitions were automatically aligned. If the standard resources, the parts-of-speech blocker and the MeSH-dictionary were used or not were instead altered in the 8 possible ways.

**Table 7:** Automatic word alignment results from batch 6

Batch	Run	Resources																	Alignment							Result		
												
		Static			Statistic							Dynamic																
		
		Stan	POS	MeSH	Statistic partition							Training partition							Test partition							Recall	Precision	F-score
								
					2	3	4	5	6	7	8	2	3	4	5	6	7	8	2	3	4	5	6	7	8			
6	1																		X	X	X	X	X	X	X	0.29	0.38	0.33
6	2											X	X	X	X	X	X	X	X	X	X	X	X	X	X	0.77	0.76	0.76
6	3				X	X	X	X	X	X	X								X	X	X	X	X	X	X	0.58	0.61	0.59
6	4				X	X	X	X	X	X	X	X	X	X	X	X	X	X	X	X	X	X	X	X	X	0.80	0.77	0.78
6	5	X	X	X								X	X	X	X	X	X	X	X	X	X	X	X	X	X	0.79	0.77	0.78
6	6	X	X	X	X	X	X	X	X	X	X								X	X	X	X	X	X	X	0.72	0.69	0.70
6	7	X	X	X	X	X	X	X	X	X	X	X	X	X	X	X	X	X	X	X	X	X	X	X	X	0.81	0.77	0.79

Recall, precision and F-score from the automatic word alignment when resources for the automatic word alignment were generated from all partitions and all partitions were automatically aligned. If the static resources, statistical resources and training resources were used or not were altered according to the table.

### Dictionary creation

The grouping into base forms of the unique candidate term pairs resulted in 29,435 term pairs. Of these pairs 19,087 were included in the list of correct term pairs from the earlier study [2] and the rest of the pairs were manually categorised. The results of the whole process are included in Table 8.

**Table 8:** Candidate term pairs evaluation results

	Base forms	Inflected forms
Correct	23,737	28,342
Partly correct	4,081	4,401
Incorrect	1,617	1,691

Total	29,435	34,434

Numbers of correct, partly correct and incorrect term pairs after the manual evaluation of the candidate term pairs. Results for term pairs grouped together in their base forms and in their inflected forms are shown.

**Figure 2:**
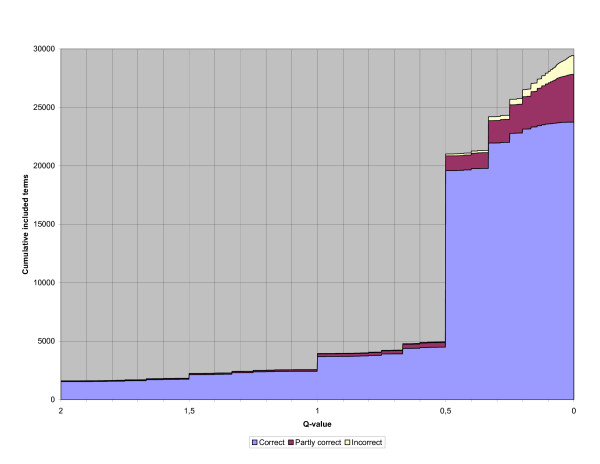
**Included term pairs per qvalue**. The cumulative number of correct, partly correct and incorrect term pairs included for a specific qvalue. (All term pairs with a qvalue equal to or greater than the actual qvalue are included.) Only qvalues equal to or smaller than 2 are included in the figure.

**Figure 3:**
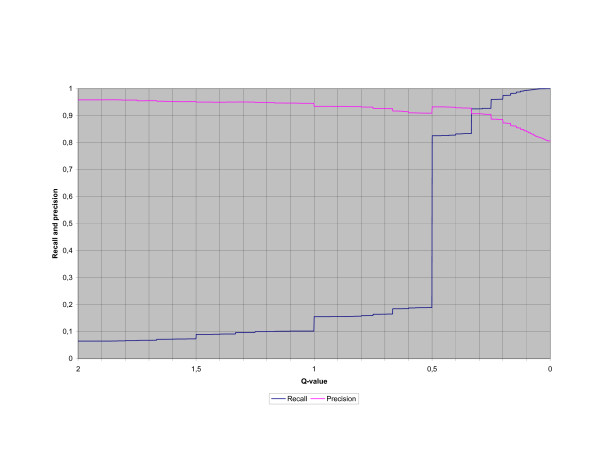
**Recall and precision per qvalue**. The recall and precision for a specific qvalue. (All term pairs with a qvalue equal to or greater than the actual qvalue are included.) Only q values equal to or smaller than 2 are included in the figure.

In Figure 2 the cumulative numbers of correct, partly correct and incorrect term pairs are included for a specific qvalue and in Figure 3 the recall and precision are included for a specific qvalue. (All term pairs with a qvalue equal to or greater than the actual qvalue are included.) The figures only contain qvalues equal to or smaller than 2 because the number of included term pairs increases slowly for higher qvalues.

### Intra-rater reliability

The results of the intra-rater reliability study are presented in Table 9 as F-score.

**Table 9:** Intra-rater reliability results

Part	Number of links in original alignment	Number of links in repeated alignment	Number of mutual links	F-score
2	618	619	561	0.91
3	280	282	257	0.91
4	136	139	131	0.95
5	2,416	2,446	2,314	0.95
6	881	883	833	0.94
7	94	99	89	0.92
'8	492	478	464	0.96

Number of links in original alignment, number of links in repeated alignment, number of links mutually included in both alignments and F-score.

The manual inspection of the word alignment variability showed that the most common differences are if a sequence of words is treated as a single name or if the words are split up in a modifier and a name. (The style guide for the manual word alignment is provided in Appendix3.) An example of a single name could be 'Down's syndrome' and of a modifier and a name could be 'malignant neoplasm', but in the problematic cases the border between single names or modifiers and names were fuzzier than in these examples. Because of the repetitive structure of terminology system rubrics, the word alignment differences were also repetitive.

## Discussion

### Partition characteristics

The results from batch 1–4 can be used to evaluate the quality of resources generated from different partitions and the results when the resources are used to automatically align different partitions. The results can also be used to evaluate similarities and differences within and among the partitions. These characteristics are important to know for word alignment purposes but also contain useful information about the terminology systems themselves.

#### Partition 2 (MeSH, part of)

Partition 2 is the partition with lowest number of words per rubric of the analysed partitions and could therefore be expected to be uncomplicated to automatically word align. This partition, together with partition 5, also has the highest number of unique words per rubric. Resources generated from this partition could therefore be expected to improve the automatic word alignment results more than other resources.

In Batch 1, partition 2 has the best recall and precision in the CfStatistical configuration, which means that it is the simplest partition to align with only statistical resources from the partition. The reason is probably the short rubrics and a high degree of repetitive structure. The configurations CfStatisticalStatic and CfStatisticalStaticTraining increase the recall and precision, but the improvements are small in comparison with the other partitions. The recall and precision for the CfStatisticalStaticTraining configuration are therefore unexpectedly one of the lowest in this batch. The probable reason is that static resources and human training do not add much knowledge of the partition that can not be acquired from statistical resources.

In batch 4, partition 2 has a lower recall and precision than most other partitions. When resources are built from a union of the partitions, the main part is generated from clinical medical terminology systems, but partition 2 is part of a bibliographic medical terminology system. Thus, one explanation of this result can be that the content and structure differ between clinical and bibliographical terminology systems.

#### Partition 5 (ICD-10, except chapter 2 level 4)

In batch 2, most partitions give similar results despite their different sizes. Partition 5 is the exception and gives better recall and precision in all the three configurations. In batch 3, the addition of partition 5 gives the largest increase in recall and precision. These results indicate that partition 5 covers the medical domain, represented as the union of all partitions, best of the single partitions. This is a reasonable result, since partition 5 contains 29 percent of the English and 28 percent of the Swedish unique words in the union of all partitions. Partition 5 contains the main parts of ICD-10, and ICD-10 is the only disease classification included that is intended to cover the complete medical domain. For the other partitions, contents and structures are probably more partition-specific and not straightforward to generalise to other partitions.

#### Partition 4 (KSH97-P) and 5 compared

In batch 1 and batch 2, partition 5 shows better results than partition 4. In batch 3, partition 5 increases the recall and precision notably more than partition 4 despite the fact that partition 4 is added to the accumulated resources before partition 5. Partition 5 contains the main part of ICD-10 and partition 4 contains KSH97-P, which is based on ICD-10. Thus they have similar content and structure, but partition 5 has eleven times more rubrics, four times more unique English words and five times more unique Swedish words than partition 4. The difference in sizes is therefore the probable reason why partition 5 gives better results than partition 4. However, the difference in size is relatively large compared to the differences in results and therefore using resources generated from a larger volume of rubrics might not be the most efficient way to improve the results.

#### Partition 7 (ICD-10, chapter 2 level 4)

In batch 1 and batch 4, partition 7 is the most difficult partition to automatically word align. This partition consists of the part of ICD-10 that was separated from the main part because the Swedish rubrics mostly start with a phrase that does not exist in the English rubrics. The hypothesis was that this difference reduces the quality of the generated resources and the results found in batch 1 and batch 4 supports this hypothesis. This is a reason for trying to identify translation inconsistencies before the word alignment starts.

The small size of this partition leads to a small training set, but also that there are few unique words to automatically align. Therefore the inconsistent start phrases are still the most probable reason for the bad results.

#### Partition 8 (NCSP, chapter N)

In both batch 1 and batch 4, partition 8 gives the highest recall and precision for the configuration CfStatisticalStaticTraining, which were not expected. However, this partition is from one single chapter of NCSP and compared to the other partitions it has an extremely low number of unique words per rubric. The many repetitions of the same words in the rubrics gives many possibilities to discover the correspondences of the words when the resources are generated and this is the most probable reason for the good results.

#### Partition 7 and 8 compared

Partition 7 and 8 give small increases in recall and precision in batch 3. Because of the low quality of the translation of these partitions we had a hypothesis that they would even decrease the recall and precision when they were added. However, they are relatively small–both according to the number of rubrics and especially according to the number of unique words–compared to the other partitions, which might reduce the effect of the lower quality.

#### Partition 3 (ICF), 5, 6 (NCSP, except N) and 8 compared

Partitions 3, 5, 6 and 8 give good recall and precision in batch 1 and batch 4. These partitions contain the whole or parts of the terminology systems ICF, ICD-10 and NCSP. These terminology systems have been built and translated during large systematic projects and their main parts can therefore be assumed to have repetitive structure in the rubrics and similar structure in the original and translated rubrics, which make them easier to automatically align, especially after manual training.

All of these terminology systems are also clinical terminology systems and when resources are generated from a union of the partitions is it therefore reasonable that they have similar results in batch 4.

### Automatic word alignment characteristics

In this section the questions in the section Automatic word alignment are answered. One purpose of batch 1–4 was to enable analysis of the performance of the automatic word alignment when different types of resources are used on different partitions. Batch 5 and 6 were mainly used for evaluation of the performance of the automatic word alignment when different types of resources are used on the union of the partitions. These characteristics are important when selecting methods for automatic word alignment.

#### Batch 1 and 2

In batch 1 and 2 the recall and precision generally increase more between the configurations CfStatistical and CfStatisticalStatic than between CfStatisticalStatic and CfStatisticalStaticTraining, but there are still notable differences between CfStatisticalStatic and CfStatisticalStaticTraining. Statistical resources and static resources are generated automatically but training resources are generated by manual word alignment. The costs of generating training resources are therefore considerably higher than for the other resources, but the improvement in results probably justifies the costs. It is also probable that both the statistical resources and training resources partly contain the same information.

#### Batch 2

In batch 2 all partitions except partition 5 have similar recall and precision despite the large difference in number of rubrics and number of unique words in the partitions used to build the resources. This indicates that the size of the partition used for building the resources only partly influences the results. There is probably much information in common between resources generated from the different partitions.

The results of the automatic word alignments in batch 2 are not as good as in batch 1 and 4. A probable reason is the configuration in batch 2, because batch 2 is the only batch where the automatic word alignment to a large extent aligns the partitions without resources generated from the current partition. This means that resources generated from a single partition, possibly except partition 5, are not easy to generalise to other partitions.

#### Batch 3

For all the three configurations, recall and precision increase for each added partition. When a partition is added more resources are available for the automatic alignment, and for each added partition, a larger part of the used resources are generated from the partitions that are being aligned. Therefore is it a sensible result that the recall and precision become better for each added partition.

#### Batch 4

In batch 4 the resources are generated from all partitions instead of only some partitions and this is a probable reason why this batch gives better results than batch 1. However, compared to batch 1 the improvements are small except for partition 4 and 7, which have the fewest number of rubrics. More partitions therefore seem to give better results and partition 4 and 7 are so small that widely useful resources are impossible to generate from them.

#### Batch 5

In all runs in batch 5, recall and precision differ only marginally. When both statistical resources and training resources exist, there is not any genuine need to use standard resources, the parts-of-speech blocker and the MeSH-dictionary.

One reason why the static resources do not improve the results may be that they contain information that overlaps the information in the training resources.

Regarding the parts-of-speech blocker it depends on the tagging of the rubrics done by the parts-of-speech tagger. The tagger is constructed for natural language while rubrics in medical terminology systems often contain words or rubrics in Latin and Greek, which sometimes cause the tagger to tag words incorrectly. This is a probable reason why the parts-of-speech blocker does not improve the results.

#### Batch 6

Batch 6 shows that if no resources are used for the automatic alignment the recall and precision are very low, which are expected. The reason why the results are not even lower is the heuristics, which are still applied.

The use of only statistical resources gives low recall and precision, but use of only training resources gives recall and precision close to the result when all kinds of resources are used. Using both statistical resources and training resources gives a small improvement compared to only using training resources.

When static resources are added to the three configurations above the same pattern appears in the three configurations with static resources as in the three configurations without static resources. The main difference is the large increase in recall and precision between only statistical resources and static resources together with statistical resources. Static resources and training resources give clearly better results than static resources and statistical resources. The improvements of using both static resources, statistical resources and training resources are also small compared to only using training resources.

The information in the statistical resources must then mainly be a subset of the information in the training resources. Therefore, the training resources are more important than statistical resources, and the training resources can be used without statistical resources with only a small worsening of the results. However, statistical resources can be built automatically while training resources are built from manually aligned texts. Statistical resources are therefore less expensive to build, which is an argument to still consider statistical resources to improve the performance.

#### Batch 5 and 6

A comparison of batch 5 and batch 6 gives that if training resources exist and are used then adding standard resources, parts-of-speech blocker and MeSH-dictionary only give small improvements of the results. However, if training resources do not exist then statistical resources together with static resources can give a decent result.

### Generated dictionary

The generated English-Swedish dictionary contains 23,737 term pairs in base forms. This is less than the dictionary generated in the earlier study [2], which contained 30,997 term pairs, but the dictionaries are generated with different set-ups. In the earlier study both base forms and inflected forms of the term pairs were included in the dictionary, but in the current study only base forms are included. Partition 1 was in the earlier study also aligned and included in the generated dictionary, but in the current study partition 1 was used as a dictionary resource in the word alignment process and therefore not included in the generated dictionary. A more appropriate number of term pairs to compare with the earlier study are therefore the number of correct term pairs in their uninflected forms, 28,342, plus the size of partition 1, 13,514, which is 41,856. This is a substantially better result than in the earlier study.

#### Using qvalues

Instead of the labour-intensive manual categorisation into correct term pairs, partly correct term pairs and incorrect term pairs for the dictionary generation, it is possible to use the qvalues of the candidate term pairs. In that case, only a sample of the candidate term pairs are manually categorised and a figure similar to Figure 2 is drawn according to the categorisation of the sample. The figure is then used to find a cut-off qvalue where a high proportion of the candidate term pairs with a higher qvalue are assumed to be correct and similarly a high proportion of the candidate term pairs with a lower qvalue are assumed to be incorrect. If a high cut-off qvalue is selected, then a large set of correct term pairs are included, but there will also be a large number of correct term pairs that are excluded. If a low cut-off qvalue is selected many incorrect term pairs are included, but few correct term pairs are excluded. An important observation is that if the data for the figure are based on a categorised sample of the candidate term pairs instead of a categorisation of all candidate term pairs, the figure is more coarse-grained and the cut-off qvalue is harder to select than in this study.

If a precision of around 0.9 is not too low then, according to Figure 2, the cut-off qvalue 0.25 can be selected and the resulting recall will be 0.96, which is a high recall. If instead a precision of 0.95 or higher is required a high cut-off qvalue has to be selected and the resulting recall will be equal to or less than 0.10, which is in most cases is a too low recall.

One problem with input materials like the terminology systems used in this study is that they result in many term pairs which only occur once. This is clearly indicated in Figure 2 and Figure 3 where it is obvious that many candidate term pairs have the qvalue 0.5 independently of whether they are categorised as correct, partly correct or incorrect. The reason for this is exactly that these term pairs only occur once in the input data as well as in the resulting alignments.

### Intra-rater reliability

The F-score in the intra-rater reliability study varied between 0.91 and 0.96 for the different partitions. The manual word alignment used for generation of training resources can therefore be expected to have been carried out reliably.

The number of differences when a sequence of words is treated as a single name in one alignment and a modifier and a name in another alignment could maybe have been fewer with a slightly modified study. Two possible modifications could have been

1. the word alignment style guide for the manual word alignment could have stated the differences between the two alternatives more clearly

2. the manual aligner could have been given more training time before aligning the material used in the study

It is however unclear how few the differences can be, because in reality there is no clear cut division between a sequence of words as a single name or a modifier and a name.

## Conclusion

More resources give better results in the automatic word alignment, but some resources only give small improvements. The most important type of resource is training, which alone gives nearly as good results as the union of all types of resources. The union of static resources and statistical resources gives not as good result as only training resources, but can be built without manual word alignment.

Partition 5, which contains the major parts of ICD-10, is the partition that alone generates the best resources for the automatic word alignment of the union of the partitions. A probable reason is the sheer size of ICD-10 and that it includes a large portion of all unique words. The diagnostic domain of ICD-10 might also be the best one to cover the whole domain of the used terminology systems.

Resources generated from MeSH seem to be more difficult than other resources to generalise to other terminology systems. There is a difference in content and structure between MeSH, which is a bibliographic medical terminology system, and the other terminology systems, which are clinical medical terminology systems. This difference is a probable reason why resources generated from MeSH are more difficult to generalise.

## List of abbreviations

ICD-10: International Statistical Classification of Diseases and Related Health Problems, Tenth Revision;

ICF: International Classification of Functioning, Disability and Health;

KSH97-P: Primary Health Care Version of The International Statistical Classification of Diseases and Related Health Problems (In Swedish Klassifikation av sjukdomar och hälsoproblem 1997 – Primärvård);

MeSH: Medical Subject Headings;

NCSP: NOMESCO Classification of Surgical Procedures;

NLM: United States National Library of Medicine;

NOMESCO: Nordic Medico-Statistical Committee;

WHO: The World Health Organization.

## Competing interests

Author Magnus Merkel is co-owner of the company Fodina Language Technology AB, which holds the rights to commercial exploitation of the ITools suite.

## Authors' contributions

MN collected the data and divided the data into partitions, planned the experimental set-up, did the manual training in ILink and the manual categorisation in IViev, analysed the data and wrote the article except for the sections 'Word alignment' in the 'Background' and 'Alignment tools' and 'Ranking and filtering candidate term pairs' in the 'Methods'.

MM configured, ran and calculated the results from the experiments in ITools, substantially contributed in the design of the study, wrote the sections 'Word alignment' in the 'Background' and 'Alignment tools' and 'Ranking and filtering candidate term pairs' in the 'Methods' and substantially contributed to drafting and revising the remaining parts of the manuscript.

HP and HÅ substantially contributed in the design of the study, and in drafting and revising the manuscript.

All authors read and approved the final manuscript.

## Appendix1: Glossary

**batch **: A collection of automatic word alignment runs where the configurations are tailored to answer a specific question

**candidate term pair **: An automatically aligned term pair which is the candidate to be included in the English-Swedish medical dictionary.

**correct term pair **: An automatically aligned term pair which a manual evaluation has classified as correct and will be included in the English-Swedish medical dictionary.

**incorrect term pair **: An automatically aligned term pair which a manual evaluation has classified as incorrect.

**MeSH-dictionary **: A dictionary used as a static resource in the word alignment. The dictionary consists of all rubric pairs in MeSH which have one word in either English or Swedish.

**partly correct term pair **: An automatically aligned term pair which a manual evaluation has classified as partly incorrect.

**partition **: A set of term pairs which contains all of or a part of a terminology systems' term pairs.

**parts-of-speech blocker **: A blocker that filters out any alignment that matches a certain predefined parts-of-speech pattern that is deemed to be unwanted.

**rubric **: The English or Swedish preferred label associated with a code in a terminology system.

**run **: An automatic word alignment run with a specific configuration.

**standard resources **: Standardised resources which contain two static standard English-Swedish dictionaries and patterns for common parts-of-speech correspondences.

**static resources **: The union of the standard resources, the parts-of-speech blocker and the MeSH-dictionary.

**statistical resource **: A dictionary generated automatically through statistical analysis of parallel rubrics.

**term pair **: An English word or sequence of words and a Swedish word or sequence of words which have been grouped together by manual or automatic alignment.

**training resource **: A resource built from manual word alignment from a sample of parallel rubrics.

## Appendix2: Used terminologies

### ICD-10

International Statistical Classification of Diseases and Related Health Problems, Tenth Revision, ICD-10, is provided by WHO[14]. ICD-10 is a statistical classification divided into 21 chapters with a broad and general coverage of mainly diseases and health related problems but also external causes and factors influencing health[14]. The Swedish National Board of Health and Welfare is responsible for the Swedish translation[15].

### ICF

International Classification of Functioning, Disability and Health, ICF, is provided by WHO[16]. ICF is aimed to be a framework for describing health and health related states. Its four chapters cover the areas 'Body functions', 'Body structures', 'Activities and participation' and 'Environmental factors'[16]. The Swedish National Board of Health and Welfare is responsible for the Swedish translation[17].

### MeSH

Medical Subject Headings, MeSH, is provided by the United States National Library of Medicine[18]. MeSH is a controlled vocabulary mainly used for indexing articles from 4,800 biomedical journals, but also used for indexing other kinds of resources, like books, documents and audio-visual material. The used version is the year 2003 version. The library at Karolinska Institutet is responsible for the Swedish translation[19].

### NCSP

NOMESCO Classification of Surgical Procedures, NCSP, is provided by the Nordic Medico-Statistical Committee (NOMESCO)[20]. NCSP is a statistical classification of surgical procedures for the Nordic countries. Its 15 main chapters consist of surgical procedures arranged by functional and anatomic body systems, the 4 subsidiary chapters contain therapeutic and investigative procedures and the supplementary chapter contains qualifiers to the other chapters[20]. The version used here is the year 2004 revision 1. The Swedish National Board of Health and Welfare is responsible for the Swedish translation[21].

### KSH97-P

Primary Health Care Version of the International Statistical Classification of Diseases and Related Health Problems, KSH97-P, is provided by the Swedish National Board of Health and Welfare[22]. KSH97-P is a statistical classification derived from the Swedish version of ICD-10 and the coverage is the common diseases and health related problems in the Swedish primary healthcare. Parts of its rubrics are identical to rubrics in ICD-10, while other rubrics are aggregates for rubrics in ICD-10. The English translation is made available by the Swedish National Board of Health and Welfare [23].

## Appendix3: Word alignment style guide for the manual word alignment

In the examples words that are underlined are aligned to each other and words that are strikethrough are marked as deleted.

### General rules

• Align as many words as necessary for agreement in both languages.

• Align as few words as possible with kept agreement in both languages.

• Aligned term pairs ought to be found in dictionaries.

### Word missing

If one or more words only exist in one language these words are marked as deleted.

Example *Tularaemia*

Tularemi (harpest)

Retina

Malign tumör i retina

### Name

The complete names of diseases, syndromes, procedures and similar entities are aligned to each other, but if modifiers exist they are aligned on their own. A modifier is a free-standing word, that for instance indicates state, location, cause, the method of attack or severity.

Example *Yellow Fever*

Gula febern

Malignant neoplasm of rectum

Malign tumöri i ändtarmen

Keratoconjunctivitis due to adenovirus

Keratokonjunktivit orsakad av adenovirus

Down's syndrome, unspecified

Downs syndrom, ospecificerat

DNA Polymerase III

DNA-polymeras III

### Punctuation mark and connecting word

When the punctuation mark or the connecting word is used in the same way in both languages, they divide the rubrics in parts and the parts are aligned separately.

Example *Histoplasmosis, unspecified*

Histoplasmos, ospecificerad

Cysticercosis of eye

Cysticerkos i ögat

When the punctuation mark or the connecting word is used in the same way in both languages and have the same meaning, the punctuation marks or the connecting words are aligned to each other.

Example *Histoplasmosis*,* unspecified*

*Histoplasmos*,* ospecificerad*

Mumps without complication

Påssjuka utan komplikation

When the punctuation mark or the connecting word is used in the same way in both languages but do not have the same meaning, the punctuation marks or the connecting words are marked as deleted.

Example *Respiratory failure, not elsewhere classified*

Respiratorisk insufficiens som ej klassificeras annorstädes

### Definite article

The definite article is excluded from the term if it only corresponds to the definite form of a word. The definite article is then marked as deleted.

Example *Irritation in the ear*

Irritation i örat

Irritation in the ear

Irritation i örat

### Adjective

Adjectives are aligned independently if none of the rules about names can be used.

Example *Industrial Microbiology*

Industriell mikrobiologi

### Paraphrased rubric

When the rubrics in both languages are paraphrased and there is no concordance between the single words, the rubrics are aligned to each other as a whole.

Example *Deficiency of vitamin E*

E-vitaminbrist

Lower uterine segment caesarean section

Abdominalt kejsarsnitt på istmus

## Pre-publication history

The pre-publication history for this paper can be accessed here:


